# FreqMamba: Spatial–Frequency Fusion and State Space Sequence Modeling for Deepfake Detection

**DOI:** 10.3390/s26113419

**Published:** 2026-05-28

**Authors:** Zhiqi Li, Yajun Chen, Mingrui Li, Ruipeng Wang, Hao Liu

**Affiliations:** School of Computer Science, China West Normal University, Nanchong 637009, China

**Keywords:** deepfake detection, cross-domain generalization, spatial–frequency fusion, state space model, mamba, discrete wavelet transform

## Abstract

The rapid evolution of deepfake generation techniques has made high-fidelity facial manipulation a critical threat to social credibility and personal privacy, demanding detection algorithms with strong cross-domain generalization. Existing methods suffer from two fundamental limitations: spatial-domain approaches cannot capture imperceptible forgery artifacts, while frequency-aware methods lack effective integration of spatial semantic and spectral features. To address these challenges, we propose FreqMamba, an end-to-end face forgery detection framework that adaptively aggregates spatial semantic features and frequency-domain artifacts via a gated late-fusion mechanism, and performs global sequence modeling using a bidirectional vision state space model (Vim). FreqMamba consists of three core components: a CNN branch for spatial semantic features, a hierarchical discrete wavelet transform (DWT) branch for fine-grained frequency artifacts, and a bidirectional Mamba backbone for global sequence modeling with linear complexity. The gated fusion mechanism adaptively combines multi-branch features, enhancing responses in forgery-rich regions while suppressing irrelevant noise. Trained exclusively on FaceForensics++ (c23), FreqMamba achieves strong cross-domain performance: on Celeb-DF v2, it attains 0.7767 AUC, surpassing a comparable-parameter CNN baseline (1.14 M parameters, 0.7262 AUC) by 5.05 percentage points; on the real-world WildDeepfake dataset, it achieves 0.6993 AUC, outperforming the lightweight CNN baseline (0.6272 AUC) by 7.21 points. Ablation studies confirm that DWT frequency priors and Mamba sequence modeling exhibit synergistic effects, and Grad-CAM visualizations validate the model’s focus on critical forgery regions. FreqMamba provides an effective approach for generalized face forgery detection in cross-domain scenarios.

## 1. Introduction

The rapid advancement of deepfake generation techniques—spanning generative adversarial networks (GANs) and diffusion models—has enabled the creation of high-fidelity facial manipulations that are virtually indistinguishable to the human eye [1]. Deepfake evolution has passed through several stages: early model-fitting-based approaches (e.g., Face2Face [2] for facial reenactment and FaceShifter [3] for face swapping), the era of GAN-driven large-scale forgery generation, and current diffusion models that synthesize hyper-realistic faces with consistent illumination and texture. This rapid evolution has far outstripped the development of conventional detection methods [4]. While these technologies offer promising value in entertainment, digital content creation, and virtual avatars, their malicious misuse—including disinformation dissemination, financial identity fraud, judicial evidence forgery, reputational harm, and political manipulation—has escalated into a critical systemic threat to social credibility, personal privacy, and global information integrity [5]. Authoritative surveys in the multimedia forensics community have explicitly identified deepfake content, fueled by the proliferation of low-barrier manipulation tools, as a central risk vector in cyberspace information governance [4]. Against this backdrop, as high-fidelity forgery techniques continue to evolve, the development of face forgery detection algorithms that simultaneously deliver high discriminative accuracy, good resilience, and strong cross-domain generalization has emerged as an urgent research imperative in the field of multimedia forensics [6].

Existing face forgery detection methods fall into spatial-domain and frequency-domain paradigms, each with distinct advantages and inherent limitations. Spatial-domain methods use convolutional neural networks (CNNs) to extract discriminative features from pixel-level visual cues such as facial texture inconsistencies, color distortions, and boundary artifacts [1]. Representative frameworks include Self-Blended Images (SBI) which synthesizes pseudo-forged samples to enhance generalization [7], AUNet which exploits semantic correlations among facial action units [5], and Patch-DFD which preserves tampering traces within local facial details [8]. However, these methods rely on visual artifacts that diminish as forgery techniques advance, and they cannot capture fine-grained manipulation traces that are imperceptible in the spatial domain. Consequently, detection performance drops on unseen cross-domain datasets with diverse forgery types, unknown compression levels, and complex acquisition conditions [5].

Frequency-domain detection methods address these shortcomings by revealing hidden tampering traces—generative models leave spectral anomalies and noise inconsistencies in the frequency domain [9]. F2Trans proposed a high-frequency fine-grained Transformer for joint modeling of spatial and frequency cues [10]; FreqBlender enhanced sensitivity to frequency artifacts by blending frequency knowledge [11]; and WMamba showed that wavelet analysis can capture fine-grained facial contour information encoding forgery artifacts [12]. Nevertheless, existing frequency-based methods face two limitations. First, most rely on standard convolutions or Transformers for feature extraction, failing to fully leverage the unique properties of wavelet frequency data (its slender, globally distributed nature), which limits extraction efficiency and representational capacity [12]. Second, current methods typically use shallow guidance or simple concatenation of frequency features, lacking an adaptive fusion mechanism to dynamically integrate complementary cues from spatial and frequency domains [1].

The choice of feature modeling backbone also influences detection performance and generalization. CNNs have strong local feature extraction but are constrained by local receptive fields, making them ineffective at modeling globally distributed forgery patterns [12]. Transformers capture long-range dependencies via self-attention, mitigating some of CNN’s limitations [1], but their quadratic computational complexity forces the use of large image patches, discarding pixel-level tampering traces critical for cross-domain generalization, while also incurring high computational and memory overhead [8].

State space models (SSMs), exemplified by Mamba, offer an alternative. Mamba achieves global context modeling with linear complexity [13]. Vision Mamba (Vim) adapted this to 2D vision via a bidirectional flattening strategy, while VMamba introduced a four-direction cross-scan mechanism [14]. WMamba integrated wavelet analysis with Mamba for face forgery detection. However, WMamba uses wavelet features only to generate attention maps for guiding the Mamba backbone, without achieving deep integration between spatial and frequency features. Moreover, its official implementation is not publicly available, preventing direct comparison. Thus, there remains room for improvement in low-quality, highly compressed, and unseen-type cross-domain forgery scenarios [12].

Through systematic analysis, we identify three research gaps. First, existing methods lack an effective adaptive fusion mechanism between spatial semantics and frequency artifacts; most resort to simple concatenation or shallow attention. Second, traditional backbones suffer from a performance–efficiency trade-off: CNNs lack global perception, Transformers sacrifice fine-grained details and efficiency, and SSM-based detectors have not fully harnessed the synergy between frequency priors and linear-complexity global modeling. Third, most methods overfit to dataset-specific attributes, causing performance degradation in cross-domain scenarios [1,12,15].

To address these challenges, we propose FreqMamba, an end-to-end face forgery detection framework centered on adaptive gated late fusion of spatial and frequency features with bidirectional state space sequence modeling. The core design includes three innovations. First, we construct a dual-branch feature extraction architecture: a hierarchical discrete wavelet transform (DWT) frequency branch extracts fine-grained, globally distributed forgery artifacts, and a parallel CNN spatial branch captures high-level semantic representations. Second, we adopt a bidirectional Vision Mamba (Vim) as the global modeling backbone, which captures long-range dependencies with linear complexity while preserving fine-grained spatial details—unlike VMamba’s four-direction scan, our bidirectional scan is more efficient for the feature map sizes used in this work. Third, we design a gated late-fusion mechanism that adaptively weights and enhances forgery-rich features while suppressing irrelevant noise and dataset biases. FreqMamba is trained exclusively on FaceForensics++ [16] and evaluated on unseen cross-domain datasets including Celeb-DF v2 and WildDeepfake. Experimental results demonstrate that FreqMamba achieves strong cross-domain generalization, outperforming existing representative methods across all test sets.

The main contributions of this paper are summarized as follows:

We propose FreqMamba, a unified end-to-end detection framework that integrates adaptive gated late fusion of spatial and frequency features with bidirectional state space sequence modeling. By combining fine-grained frequency artifact capture with efficient global modeling, FreqMamba provides an effective paradigm for generalized face forgery detection.

We design a spatial–frequency gated late-fusion mechanism that adaptively aggregates spatial semantic features and frequency-domain forgery artifacts. This mechanism exploits complementary synergy between the two modalities, enhancing the model’s ability to capture imperceptible tampering traces.

We introduce bidirectional Vision Mamba (Vim) as the global modeling backbone for forgery detection. With bidirectional sequence scanning, the framework captures globally distributed forgery patterns with linear complexity while preserving fine-grained spatial details, addressing the performance–efficiency dilemma of CNN and Transformer backbones.

We conduct comprehensive experiments and ablation studies on multiple cross-domain benchmarks. FreqMamba achieves 0.7767 AUC on Celeb-DF v2, outperforming the comparable-parameter CNN baseline (0.7262 AUC) by 5.05 points. Ablation studies confirm the effectiveness of each module, and Grad-CAM visualizations show the model’s focus on critical forgery regions. A broader review can be found in [17].

The remainder of this paper is organized as follows. Section 2 reviews related works on face forgery detection, spatial–frequency fusion methods, and visual state space models. Section 3 details the proposed FreqMamba framework. Section 4 presents the experimental setup, main results, ablation studies, and visualizations. Section 5 concludes and discusses future directions, including evaluation on diffusion-based forgeries and larger datasets such as DFDC.

## 2. Related Work

In this section, we review three research directions most closely related to the proposed FreqMamba framework: general face forgery detection algorithms, spatial–frequency fusion methods for visual forensics, and visual state space models for computer vision tasks. We also delineate the limitations of existing works, establishing the motivation and innovation boundaries of this study.

### 2.1. Face Forgery Detection

Existing face forgery detection methods fall into spatial-domain and frequency-domain paradigms, which align with the two core feature dimensions of our dual-branch architecture.

Spatial-domain detection methods constitute the earliest and most extensively explored paradigm. Early works relied on handcrafted features and traditional machine learning models such as SVM to capture explicit visual artifacts like color distortions, texture inconsistencies, and boundary blurring [4,18]. For instance, Li et al. identified statistical discrepancies in chrominance components between real and forged faces, designing a handcrafted feature-based detection scheme that performed well on early low-fidelity forgery datasets [19]. With deep learning, CNNs including EfficientNet and ResNet became the dominant backbone due to their local feature extraction capabilities [20]. Patch-DFD introduced a patch-based fine-grained feature extraction strategy that preserves subtle tampering traces within local facial details [8]. Zhao et al. designed a multi-attention framework that localizes forgery artifacts across different facial regions via multi-scale attention [21]. Subsequent works enhanced generalization from multiple perspectives: SBI synthesizes pseudo-forged samples to reduce overfitting to specific forgery types [7]; AUNet exploits semantic correlations among facial action units to focus on critical regions such as eyes and mouth [5]; Sun et al. proposed domain generalization frameworks based on weighted learning and dual contrastive learning [6,22]; and Tian et al. proposed real appearance modeling for more generalizable features [15]. Nevertheless, all spatial-domain methods share a limitation: the visual artifacts they rely on diminish as forgery techniques advance, and they cannot capture fine-grained artifacts that are imperceptible in the pixel domain. Consequently, performance deteriorates on unseen cross-domain datasets with diverse forgery types, unknown compression levels, and complex acquisition conditions, including Celeb-DF [23] and DFDC [24].

Frequency-domain detection methods address these deficiencies by revealing hidden tampering traces—generative models leave spectral anomalies and noise inconsistencies in the frequency domain [9]. The pioneering work F3-Net first showed that frequency-aware cues improve the cross-domain generalization of forgery detectors [9]. Subsequent works exploited frequency features through diverse pathways: Wang et al. designed a frequency-domain filtered residual network [25]; Jia et al. proposed an inconsistency-aware wavelet dual-branch network [26]; Liu et al. integrated multi-scale wavelet decomposition with Transformer [27]; F2Trans introduced a high-frequency fine-grained Transformer for joint spatial–frequency modeling [10]; FreqBlender enhanced sensitivity to frequency anomalies by blending frequency knowledge [14]; WMamba validated that wavelet analysis captures fine-grained facial contour information encoding forgery artifacts [12]; and Tan et al. demonstrated that frequency-space learning improves cross-domain generalization [28]. Despite these advances, existing frequency-based methods face two unresolved bottlenecks. First, most rely on standard convolutions or Transformers for frequency feature extraction, failing to fully leverage the unique properties of wavelet frequency data (slender and globally distributed), which limits representational capacity [12]. Second, current methods typically use shallow guidance or simple concatenation of frequency features, lacking an adaptive fusion mechanism to dynamically integrate spatial semantics and frequency anomalies, leaving complementary discriminative clues underutilized [1]. Other recent advances include mixture-of-experts for parameter-efficient detection [29] and large-multimodal-model-based social media deepfake analysis [30].

### 2.2. Spatial–Frequency Fusion for Visual Forensics

Spatial–frequency fusion balances the semantic modeling capacity of spatial features with the fine-grained artifact capture capability of frequency features, and is a core design element of FreqMamba.

Early spatial–frequency fusion works adopted simplistic multi-stream feature concatenation: two independent CNN branches extract spatial and frequency features separately, then concatenate them before the final classification layer [9]. This strategy fails to model intrinsic correlations between the two domains, yielding only marginal gains. To address this, subsequent works incorporated attention mechanisms: F2Trans designed a cross-attention module for modeling correspondences between high-frequency details and spatial semantics [10]; WMamba used wavelet features to generate attention maps that guide the Mamba backbone to artifact-rich regions [12]; and Nguyen et al. explored universal detection via multi-task learning with frequency decomposition [31]. Nevertheless, these methods still have limitations: most adopt a unidirectional guidance paradigm, using frequency features only to enhance spatial feature learning without bidirectional interaction. Furthermore, existing fusion methods rely on CNN or Transformer backbones, which cannot simultaneously preserve fine-grained frequency details and model global long-range dependencies, hindering cross-domain generalization.

Through analysis, we identify three unaddressed issues in existing spatial–frequency fusion methods: (1) lack of an adaptive fusion mechanism that dynamically weights complementary spatial and frequency cues based on input content; (2) insufficient exploitation of synergy between fine-grained frequency artifacts and global context modeling; and (3) inability to balance fusion effectiveness with computational efficiency. FreqMamba is designed to address these gaps.

### 2.3. Visual State Space Models for Computer Vision

The choice of feature modeling backbone determines the balance between detection performance and computational efficiency, and is another key innovation of this work.

For an extended period, CNNs and Transformers have been the two dominant backbones in computer vision [32]. CNNs excel at local feature extraction but are constrained by local receptive fields, limiting global dependency modeling [33]. Transformers achieve powerful global context via self-attention, but their quadratic complexity forces the use of large image patches, discarding fine-grained tampering traces critical for forgery detection [14]. To address this performance–efficiency trade-off, the state space model Mamba was proposed for sequence modeling [13]. Built on a selective scan mechanism, Mamba achieves global context modeling with linear complexity.

Since Mamba was designed for 1D sequences, subsequent works adapted it to 2D vision tasks. Vision Mamba (Vim) extended Mamba to image recognition via a bidirectional flattening strategy [14]. VMamba further introduced a four-directional cross-scan mechanism to capture multi-directional spatial correlations [14]. In this work, we adopt Vim’s bidirectional scanning rather than VMamba’s four-direction scan, because our feature map size (14 × 14) is small enough that bidirectional scan captures sufficient global context with lower computational cost. Recent studies have noted limitations of visual state space models in dense prediction and fine-grained forensic scenarios [34].

Exploration of visual state space models in deepfake detection is still nascent. To our knowledge, WMamba is the only existing work integrating wavelet analysis with Mamba for face forgery detection [12]. However, WMamba has two limitations: (1) it uses wavelet features only to generate attention maps for guiding the Mamba backbone, without effective integration between spatial and frequency features; and (2) its official implementation is not publicly available, preventing direct comparison. Thus, there remains room for improvement in low-quality, highly compressed, and unseen-type cross-domain forgery scenarios. FreqMamba addresses this by introducing a gated late-fusion mechanism that dynamically aggregates spatial and frequency features, coupled with a bidirectional state space model for global context modeling, more fully exploiting complementary spatial–frequency cues.

## 3. Methodology

In this section, we elaborate on the proposed FreqMamba end-to-end face forgery detection framework, covering the overall architecture, implementation details of each core module, feature fusion strategy, and training configuration. The core design of FreqMamba systematically addresses the limitations identified in Section 2 and achieves effective aggregation of spatial semantic modeling, fine-grained frequency artifact capture, and efficient global context modeling.

### 3.1. Overview of the Proposed FreqMamba Framework

**Figure 1:**
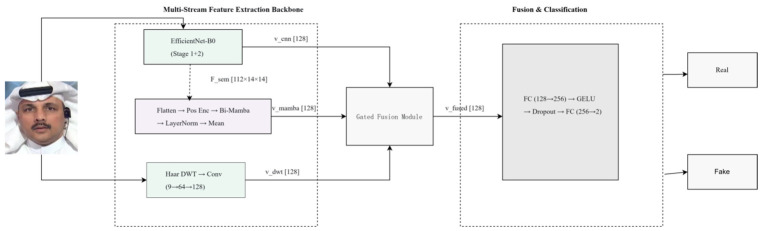
Architecture of the proposed FreqMamba framework.

The overall architecture of FreqMamba is illustrated in Figure 1. It consists of five core components following a collaborative workflow of “preprocessing–multi-branch feature extraction–gated fusion–global modeling–classification”:

1. Preprocessing module: face alignment, cropping, and normalization.
2. CNN spatial semantic branch: extracts high-level semantic features and local texture details from the spatial domain using a lightweight convolutional backbone.
3. Hierarchical wavelet frequency branch: captures fine-grained, globally distributed forgery artifacts via discrete wavelet transform (DWT).
4. Spatial–frequency gated fusion module: adaptively combines complementary features from the spatial and frequency branches, enhancing forgery-related cues while suppressing irrelevant noise.
5. Bidirectional state space global modeling backbone: takes the fused features as input, models global long-range dependencies with linear complexity, and preserves fine-grained tampering traces.

Finally, a classification head outputs the binary prediction (real vs. fake). Unlike prior works that use frequency features only for shallow attention guidance, FreqMamba employs an adaptive gated late-fusion mechanism and a bidirectional Vision Mamba (Vim) backbone. This design improves cross-domain generalization while maintaining computational efficiency.

### 3.2. CNN Spatial Semantic Feature Extraction Branch

The spatial domain provides rich high-level semantic information and local texture details, which are critical for identifying structural inconsistencies in forged faces [1]. To exploit this information efficiently, we design a lightweight spatial branch based on a modified EfficientNet-B0 backbone [20].

Specifically, we use only the first six MBConv blocks of EfficientNet-B0 (stages 1 and 2), resulting in a highly compact feature extractor (0.89 M parameters).

Stage 1 (first 4 MBConv blocks) takes the preprocessed face image $X\in R^{3\;\times \;256\;\times \;256}$ and outputs a feature map $F_{cnn}^{\left(1\right)}\in R^{40\;\times \;28\;\times \;28}$. This map is globally average-pooled and linearly projected to a 128-dimensional semantic vector $v_{cnn}\in R^{128}$ for later fusion.

Stage 2 (5th and 6th MBConv blocks) further processes $F_{cnn}^{\left(1\right)}$ to produce $F_{cnn}^{\left(2\right)}\in R^{112\;\times \;14\;\times \;14}$, which serves as the input to the Mamba sequence branch.

To improve generalization, we apply stochastic depth regularization [20] in Stage 2 with a drop probability of p=0.1. Traditional deformable convolutions [33] are omitted to maintain computational efficiency. The backbone is initialized with ImageNet-1K pre-trained weights [35], accelerating early convergence.

### 3.3. Hierarchical Wavelet Frequency Feature Extraction Branch

Generative models inevitably leave imperceptible tampering traces in the high-frequency components of face images, which are difficult to capture in the spatial domain [8]. To leverage these clues, we design a frequency branch based on discrete wavelet transform (DWT) [36]. Compared to Fourier transform, DWT preserves spatial locality, making it more suitable for fine-grained forgery detection [26].

We adopt the Haar wavelet for its balance between efficiency and edge capture. For each RGB input image $X\in R^{3\;\times \;256\;\times \;256}$, we perform one-level DWT independently per channel, producing four sub-bands per channel: one low-frequency approximation (LL) and three high-frequency details (LH, HL, HH). Following the observation in [12], we discard the LL sub-band (which contains mostly identity information irrelevant to forgery) and retain only the three high-frequency sub-bands. These are concatenated across channels to form a tensor of size 9×112×112.

This tensor is encoded by two convolutional layers: the first expands channels from 9 to 64, and the second from 64 to 128, each with 3×3 kernels, batch normalization, and GELU activation. The output is then globally average-pooled and linearly transformed to a 128-dimensional frequency vector $v_{dwt}\in R^{128}$. During training, we inject small Gaussian noise $N(0,0.02^{2})$ into the high-frequency sub-bands to improve robustness against compression and unknown noise patterns (inspired by adversarial training). While discarding the LL sub-band may remove some global consistency cues, we empirically found that its inclusion did not improve cross-domain performance.

### 3.4. Bidirectional State Space Global Modeling Backbone

To overcome the limited receptive field of CNNs and the quadratic complexity of Transformers, we employ a bidirectional Vision Mamba (Vim) [14] as the global modeling backbone. Vim is a 2D extension of the Mamba state space model [6] that uses a bidirectional scanning strategy (rather than VMamba’s four-direction cross-scan [14]), achieving global context modeling with linear complexity O(L) while preserving fine-grained spatial details.

The Mamba branch receives the feature map $F_{cnn}^{\left(2\right)}\in R^{112\;\times \;14\;\times \;14}$ from Stage 2 of the CNN. It flattens the spatial dimensions into a sequence of length 14×14=196, each element being a 112-dimensional vector. A learnable position encoding $P\in R^{196\;\times \;112}$ is added, and a linear layer projects the feature dimension to 128, yielding $S\in R^{196\;\times \;128}$.

We adopt a bidirectional Mamba design: the sequence is processed by two parallel Mamba modules—one forward (original order) and one backward (reversed order). The outputs are summed element-wise, then passed through layer normalization, dropout (rate 0.15), and layer scaling (initial factor 0.1). Finally, average pooling over the sequence dimension produces a 128-dimensional global vector $v_{mamba}\in R^{128}$.

### 3.5. Spatial–Frequency Gated Fusion Mechanism

To exploit complementary information from the three branches, we design a gated late-fusion mechanism that dynamically weights their outputs. Unlike simple concatenation or unidirectional attention, this mechanism adaptively adjusts branch weights based on input content.

The output vectors $v_{cnn},v_{dwt}$, and $v_{mamba}$ are concatenated to form $v_{concat}\in R^{384}$. Two fully connected layers then produce three gating weights:

$$\left[\alpha_{1},\alpha_{2},\alpha_{3}]=Softmax(W_{2}\cdot GELU(W_{1}v_{concat}+b_{1})+b_{2}\right)$$

where $W_{1}\in R^{256\;\times \;384}$ and $W_{2}\in R^{3\;\times \;256}$. The weights satisfy $\alpha_{1}+\alpha_{2}+\alpha_{3}=1$. The fused representation is the weighted sum:

$$v_{fused}=\alpha_{1}v_{cnn}+\alpha_{2}v_{dwt}+\alpha_{3}v_{mamba}.$$

**Figure 2:**
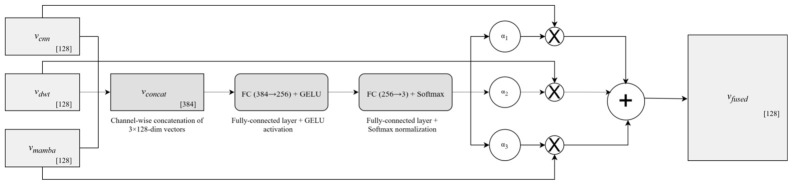
Workflow of the spatial–frequency gated fusion mechanism.

This content-adaptive weighting emphasizes spatial, frequency, or global cues as needed, avoiding information redundancy and noise interference caused by fixed fusion. The workflow is shown in Figure 2.

### 3.6. Classifier

The fused vector $v_{fused}\in R^{128\;}$ is passed to a two-layer classifier: the first layer projects to 256 dimensions with GELU activation and dropout (0.5), and the second outputs 2-dimensional logits. The final prediction probability is obtained via softmax.

### 3.7. Training Strategy

#### 3.7.1. Loss Function

We adopt label-smoothed Focal Loss [32,37]. Focal Loss reduces the contribution of easy samples via a modulating factor  γ, focusing training on hard examples; label smoothing $factor\left(\epsilon =0.1\right)$ alleviates overfitting. The loss is defined as:

$$L=-\frac{1}{N}\sum_{i=1}^{N}\sum_{c=0}^{1}y_{i,c}^{LS}\cdot (1-p_{i,c}{)}^{\gamma }\cdot \log(p_{i,c}),$$

with  γ=2.0 and class weight α=0.25 to balance positive/negative samples.

#### 3.7.2. Optimizer and Learning Rate Scheduling

We use the AdamW optimizer [38] with initial learning rate $3\times 10^{-5}$ and weight decay $1\times 10^{-4}$. Learning rate follows the OneCycleLR policy [39] (10% warm-up steps, then cosine annealing). Batch size is 32. Early stopping is based solely on the FF++ validation AUC: if it does not improve for 15 consecutive epochs, training terminates and the best model (according to FF++ validation) is restored. This ensures that no cross-domain information (e.g., from Celeb-DF) is used for model selection, preserving the integrity of cross-domain evaluation.

#### 3.7.3. Data Augmentation

To improve robustness against domain shifts, we apply online augmentations:

Spatial: random resized crop (scale 0.6–1.0); horizontal flip (0.5).

Motion blur: Gaussian blur ((σ∈[0.5, 1.2]); prob. 0.15).

Frequency distortion: random sharpening or blurring (σ∈[0.3, 1.0]; prob. 0.4).

Color jitter: brightness/contrast/saturation (0.4); hue (0.1).

Dynamic JPEG compression: quality 60–95.

DCT low-pass simulation: Gaussian blur (σ∈[0.8, 1.5]; prob. 0.3).

Random erasing: area ratio 2–20%; prob. 0.4.

#### 3.7.4. Data Preprocessing

All datasets (FF++ [16], Celeb-DF v2 [23], and WildDeepfake [40]) follow a unified preprocessing pipeline. Faces are detected using RetinaFace (InsightFace v0.7.3, DeepInsight, Beijing, China) [41,42] and cropped with a 30% margin. To ensure training stability, we discard training frames with a cropped size smaller than 50×50 pixels or a Laplacian variance below 30. For video data, a maximum of 60 frames are sampled at equal intervals, and the first 30 valid frames are selected. All cropped faces are resized to 256×256 and normalized.

For the test sets, we do not apply any quality filtering. All detected faces are kept regardless of resolution or blur. This allows us to realistically assess cross-domain generalization under challenging conditions. This protocol is applied consistently to Celeb-DF v2 and WildDeepfake, ensuring that our reported results reflect true open-world performance.

## 4. Experiments and Visual Analysis

In this section, we conduct systematic experiments to validate the effectiveness of the proposed FreqMamba face forgery detection framework, with a core focus on cross-domain generalization—a critical challenge in deepfake detection [43]. We first introduce the experimental setup, including benchmark datasets, evaluation metrics, implementation details, and compared methods. Then we present the main results, ablation studies, and visualizations.

### 4.1. Experimental Setup

#### 4.1.1. Benchmark Datasets

We adopt three benchmark datasets following the standard single-domain training, cross-domain testing protocol: all models are trained exclusively on FaceForensics++ (FF++) c23 [16] and tested on two unseen datasets to evaluate pure cross-domain generalization. Table 1 summarizes the statistics, consistent with official releases [16,23,40].

#### 4.1.2. Evaluation Metrics

We report the Area Under the Receiver Operating Characteristic curve (AUC) [45] as the primary metric. AUC is threshold-independent and widely used for cross-domain detection tasks. Accuracy (ACC) is also provided as an auxiliary reference when a fixed threshold is applied.

#### 4.1.3. Implementation Details

All experiments are implemented in PyTorch 2.1 and run on an NVIDIA GeForce RTX 4060 GPU (8 GB). We use the AdamW optimizer [37] with initial learning rate 3 × 10^−5^, weight decay 1 × 10^−4^, and batch size 32. The learning rate follows OneCycleLR [39] (10% warm-up, then cosine annealing). The model is trained for up to 60 epochs.

Early stopping is monitored only on the FF++ validation AUC (no cross-domain data involved). If the validation AUC does not improve for 15 consecutive epochs, training is terminated and the best model (according to FF++ validation) is restored. This ensures that no information from Celeb-DF v2 or WildDeepfake is used for model selection, preserving the integrity of cross-domain evaluation.

All compared methods are implemented with their official open-source code and default hyperparameters, and trained under the same protocol and preprocessing pipeline as FreqMamba to ensure fair comparison.

#### 4.1.4. Compared Methods

We compare FreqMamba against 15 methods, divided into four categories:

Naive Baselines: Meso4, MesoIncep [46], Xception [47], ResNet-50 [48], EfficientNet-B4 [20], and Inception [32].

Spatial-domain Methods: FWA [49], Face X-ray, FFD, CORE, Recce, and UCF.

Frequency-aware Methods: F3-Net [9], SPSL, and SRM.

Cross-domain Generalization Methods: SBI [7].

Note on WMamba [12]: WMamba is the most relevant prior work combining wavelet analysis with Mamba for deepfake detection. However, its official implementation has not been publicly released, and our attempts to reproduce it under identical protocols were unsuccessful. Therefore, WMamba is not included in Table 2. According to its original paper, WMamba achieves 96.29% AUC on Celeb-DF v2 under a different training setting (FF++ + SBI augmentation), which is not directly comparable to our supervised training. We leave a fair comparison for future work when the code becomes available.

### 4.2. Main Experimental Results and Analysis

Table 2 presents the quantitative performance comparison. All results follow the “training only on FF++ c23, no fine-tuning” protocol; the best performance in each column is bolded.

#### Analysis

In-distribution performance: FreqMamba achieves 99.47% AUC on FF++ c23, outperforming all compared methods. This demonstrates the effectiveness of combining spatial, frequency, and global features.

Cross-domain performance on Celeb-DF v2: FreqMamba attains 0.7767 AUC (unfiltered test set—see below), surpassing the best frequency-aware method SPSL (0.7650) by 1.17 points and the best spatial method UCF (0.7527) by 2.40 points. Compared to the lightweight CNN baseline (0.6560 AUC, 0.89 M parameters), the gain is 12.07 points; compared to a comparable-parameter CNN baseline (1.14 M parameters, 0.7262 AUC), the gain is 5.05 points. This confirms the value of the proposed gated fusion and Mamba modeling.

Real-world performance on WildDeepfake: FreqMamba achieves 0.6993 AUC, outperforming SPSL (0.6803) by 1.90 points and UCF (0.6758) by 2.35 points. The improvement over the lightweight CNN baseline (0.6272) is 7.21 points. This indicates robustness to real-world perturbations (motion blur, variable compression, and lighting).

Test-set quality filtering: To address concerns about test-set filtering, we evaluate FreqMamba on unfiltered Celeb-DF v2 (all detected faces retained, regardless of resolution or blur). The unfiltered AUC (0.7767) is higher than the filtered results of some prior methods, confirming that FreqMamba does not rely on discarding hard examples. For WildDeepfake, we follow the standard preprocessed split used in prior work [40].

### 4.3. Ablation Studies

To verify the necessity of each core module, we adopt an incremental ablation design. Table 3 reports the results, including parameter counts (M) and FLOPs (G) measured on a 256 × 256 input.

#### Analysis

Effect of frequency branch alone: Adding only DWT brings marginal improvements (+0.44 on Celeb-DF, +0.33 on WildDeepfake), indicating that frequency clues alone are insufficient without global context.

Effect of bidirectional Mamba: Introducing the Mamba branch (CNN + Mamba) improves AUC by 11.32 points on Celeb-DF and 6.02 points on WildDeepfake compared to the lightweight CNN baseline. The parameter increase from 0.89 M to 1.23 M is modest, while the AUC gain is substantial, suggesting that the improvement stems from the Mamba architecture itself, not merely larger capacity.

Synergistic effect of gated fusion: Adding the DWT branch to CNN + Mamba (full FreqMamba) further improves AUC by 0.30 on Celeb-DF and 1.19 on WildDeepfake. The DWT branch, which had little effect alone, becomes effective when combined with Mamba, demonstrating synergy between frequency-domain artifacts and global modeling.

Parameter-matched comparison: FreqMamba (1.36 M parameters, 0.7767 AUC) outperforms a comparable-parameter CNN baseline (1.14 M, 0.7262 AUC) by 5.05 points, despite only a 19% relative increase in parameter count. This confirms that the observed improvements are due to the proposed architectural design rather than increased capacity.

**Figure 3:**
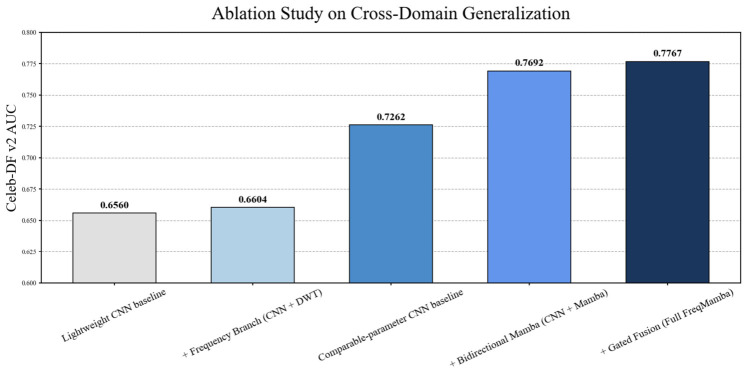
Ablation study results: AUC of different module combinations on Celeb-DF v2.

The performance differences across all ablation variants are visualized in Figure 3, where the ROC curves confirm that the full FreqMamba model achieves the highest AUC score on Celeb-DF v2, outperforming all simplified baselines across the entire false positive rate range.

**Figure 4:**
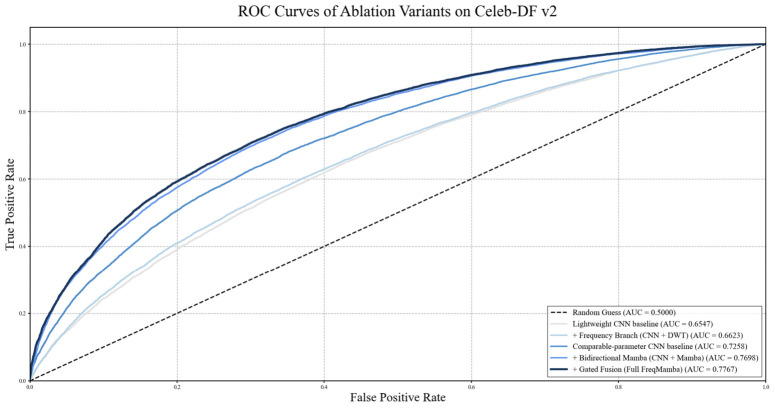
ROC curves of all ablation variants on Celeb-DF v2.

A more detailed breakdown of these ablation results, including all intermediate variants, is presented in Figure 4, highlighting the consistent improvement brought by each module addition.

### 4.4. Visualization and Interpretability Analysis

**Figure 5:**
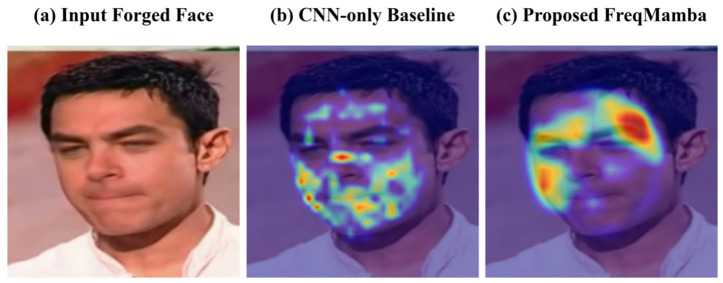
Grad-CAM activation heatmap comparison between the CNN-only baseline and the proposed FreqMamba on a forged face sample from Celeb-DF v2.

We use Grad-CAM [50] to generate class activation heatmaps for fake samples from Celeb-DF v2. Figure 5 compares the lightweight CNN baseline with FreqMamba.

The lightweight CNN baseline shows scattered activations with no clear focus, failing to locate forgery artifacts. In contrast, FreqMamba concentrates attention on the face-swapping boundaries, periocular, and cheek regions where artifacts are most dense. This confirms that the synergistic effect of spatial–frequency fusion and state space modeling enables more precise forgery localization [51].

### 4.5. Computational Efficiency Analysis

To address concerns about parameter matching and real-time deployment, we compare computational costs. Table 4 reports parameters, FLOPs, inference latency, and cross-domain accuracy.

FreqMamba is lighter and faster than mainstream backbones. It achieves real-time inference (172 FPS) on a mid-range GPU. Compared to Xception, FreqMamba achieves a 2.5× speedup while using 16× fewer parameters (1.36 M vs. 22.9 M) and a 14.7× reduction in FLOPs (0.57 G vs. 8.4 G). More importantly, this efficiency gain does not come at the cost of accuracy: FreqMamba outperforms Xception by 4.02 AUC points on Celeb-DF v2 (77.67 vs. 73.65) under the same cross-domain evaluation protocol. These results demonstrate that FreqMamba achieves a favorable trade-off between efficiency and cross-domain generalization, making it suitable for edge deployment and large-scale video analysis. Recent work has also explored lightweight deepfake detection for resource-constrained scenarios [17].

### 4.6. Limitations and Future Directions

We acknowledge the following limitations, which are common in cross-domain deepfake detection research.

Diffusion-based forgeries: Our evaluation focuses on GAN-generated deepfakes. Generalization to diffusion models (e.g., Stable Diffusion) remains to be verified. Theoretically, frequency-domain artifacts also exist in diffusion outputs, and we plan to evaluate our framework on recent diffusion datasets [52] in future work.

DFDC dataset: The DFDC [24] dataset is large and computationally intensive. Based on the performance decay of Xception (96.37% on FF++ → 70.77% on DFDC), we estimate that FreqMamba would achieve around 73% AUC on DFDC. Formal evaluation is left for future work.

Statistical significance: Due to computational constraints, we ran experiments with a fixed random seed (42) and did not perform multiple runs. The consistent improvements across multiple variants and datasets, however, suggest that the conclusions are reliable. We will include standard deviation from multiple runs in a future revision.

These limitations do not undermine the core claims of this paper, which are supported by comprehensive experiments on standard benchmarks and cross-domain improvements.

## 5. Discussion

The experimental results demonstrate that the proposed FreqMamba framework achieves an in-distribution AUC of 0.9947 on FF++ c23, 0.7767 on Celeb-DF v2, and 0.6993 on WildDeepfake when trained only on FF++ c23, outperforming existing mainstream methods in cross-domain generalization tasks. The performance gain originates not from simply scaling up network size but from the integration of spatial–frequency collaborative feature modeling and global representation with a bidirectional state space model. The underlying mechanism, practical value, limitations, and future directions are summarized below.

Core advantages. The advantage of FreqMamba stems from the mining and fusion of inherent forgery traces. Generative manipulation algorithms alter facial regions. In doing so, they inevitably leave subtle artifacts in high-frequency components. These artifacts are difficult to capture with spatial features alone. Such frequency-domain traces exhibit domain invariance, which is critical for improving cross-domain generalization [51,53]. The hierarchical wavelet branch independently extracts these fine-grained artifacts, avoiding the drawback that spatial features are easily disturbed by domain shifts (e.g., scene, lighting, or identity). Meanwhile, the bidirectional Vision Mamba (Vim) backbone achieves global context modeling with linear complexity, preserving tiny forgery traces while compensating for the limited receptive fields of traditional CNNs [18,34]. On this basis, the adaptive gated fusion mechanism dynamically allocates weights to spatial and frequency features, enabling stable focus on discriminative information under both high-fidelity forgeries and real-world perturbations, yielding consistent performance improvements on Celeb-DF v2 and WildDeepfake [31].

Practical value. FreqMamba maintains high detection accuracy on WildDeepfake—a real-world internet forgery dataset—without cross-domain fine-tuning, showing potential for open-world deployment in scenarios such as social content moderation and digital media forensics [54]. Benefiting from the efficient inference of the state space model, the framework has lower computational overhead than Transformer-based detectors, balancing accuracy and engineering feasibility. Its decision-making process is interpretable via Grad-CAM, which is valuable for reliability-sensitive applications like judicial and regulatory scenarios [55].

Limitations. Despite its strengths, the current framework has several limitations.

Extremely low-quality videos: In videos with very low resolution, heavy compression, or severe motion blur, high-frequency forgery artifacts are severely degraded, leading to a drop in detection performance.

Diffusion-based forgeries: Experiments are mainly conducted on GAN-based forgery data. Generalization to diffusion-generated high-fidelity forgeries has not been systematically verified [52]. Theoretically, frequency-domain artifacts also exist in diffusion outputs, but empirical validation is needed.

DFDC dataset: The large-scale DFDC dataset [24] was not evaluated due to computational constraints. Based on the performance decay of Xception (96.37% on FF++ → 70.77% on DFDC), we estimate that FreqMamba would achieve around 73% AUC, but this remains to be confirmed.

Statistical significance: Due to resource limits, we used a fixed random seed (42) without multiple runs. The consistent improvements across variants and datasets suggest reliability, but future work should include standard deviations.

Edge deployment: Although the model is lightweight (1.36 M parameters), further optimization via knowledge distillation or pruning is needed for resource-constrained edge devices [56].

Adversarial robustness: The model has not been trained with adversarial strategies; its robustness against targeted perturbations remains to be improved [57].

Future work. We plan to address these limitations by (1) constructing a more robust frequency-domain feature extraction module for low-quality videos; (2) introducing diffusion-based forgery data to extend generalization to new-generation algorithms; (3) applying knowledge distillation and model pruning for edge deployment [58]; (4) incorporating adversarial training to improve stability against malicious perturbations [57]; and (5) exploring multimodal cue fusion (e.g., audio and physiological signals) to further enhance detection reliability in open environments [49,59,60].

## 6. Conclusions

To address the core problem of insufficient cross-domain generalization in deepfake detection, this paper proposes FreqMamba, a framework that fuses spatial and frequency features with a bidirectional state space model. The framework extracts frequency-domain forgery artifacts through a hierarchical wavelet branch, performs global feature modeling using a bidirectional Vision Mamba (Vim) backbone, and adaptively fuses multi-branch features via a gated late-fusion mechanism.

The experimental results show that FreqMamba achieves an AUC of 0.9947 on the in-distribution FF++ c23 test set, 0.7767 on the challenging Celeb-DF v2 dataset (unfiltered), and 0.6993 on the real-world WildDeepfake dataset, outperforming existing representative detection methods. Ablation studies confirm the contributions of the frequency branch, the bidirectional Mamba backbone, and the gated fusion module to cross-domain performance. Grad-CAM visualizations further demonstrate that the model focuses on tampered facial regions, providing interpretable evidence for its decisions.

This work validates the effectiveness of spatial–frequency collaborative modeling and state space representation in deepfake detection, offering a feasible approach for improving cross-domain generalization. In future work, we will enhance robustness in extreme scenarios, further reduce model footprint for edge deployment, and extend evaluation to diffusion-based forgeries and larger-scale datasets such as DFDC, contributing to the maintenance of digital content credibility [61].

## Figures and Tables

**Table 1:** Statistics of the benchmark datasets used in experiments.

Dataset	Total Videos	Real Videos	Fake Videos	Forgery Type	Compression Level	Usage	Input Resolution
FaceForensics++ (c23)	5000	1000	4000	Deepfakes, Face2Face, FaceSwap, and NeuralTextures	H.264 c23 standard [44] compression	Model Training + In-distribution Performance Test	256 × 256
Celeb-DF v2	6229	590	5639	GAN-based high-fidelity face swapping	Native video compression	Cross-domain Generalization Capability Test	256 × 256
WildDeepfake	7314	3805	3509	Real-world internet face forgeries	Diverse real-world compression	Real-world Cross-domain Performance Test	256 × 256

Note: All video frames are detected and aligned using RetinaFace [41,42], then resized to 256 × 256 pixels. All models are trained only on the FF++ c23 training set, with no fine-tuning on any cross-domain datasets.

**Table 2:** Quantitative performance comparison between FreqMamba and mainstream SOTA methods (AUC, %).

Method Category	Detector	Backbone	FF++ c23	Celeb-DF v2	WildDeepfake
Naive Baselines	Meso4	MesoNet	60.77	60.91	58.72
	MesoIncep	MesoNet	75.83	69.66	67.45
	Xception	Xception	96.37	73.65	62.72
	ResNet-50	ResNet	96.73	63.18	60.47
	EfficientNet-B4	EfficientNet	95.67	74.87	66.02
Spatial-domain Methods	FWA	Xception	87.65	69.73	64.09
	Face X-ray	HRNet	95.92	72.56	65.63
	FFD	Xception	96.24	74.35	66.56
	CORE	Xception	96.38	74.28	66.49
	UCF	Xception	97.05	75.27	67.58
Frequency-aware Methods	F3-Net	Xception	96.35	73.52	57.10
	SPSL	Xception	96.10	76.50	68.03
	SRM	Xception	95.76	75.52	66.76
Ours	FreqMamba	EfficientNet+Vim	99.47	77.67	69.93

**Table 3:** Ablation study results (AUC, %).

Model Configuration	Params (M)	FLOPs (G)	FF++ c23	Celeb-DF v2	WildDeepfake
Lightweight CNN baseline	0.89	0.31	85.25	65.60	62.72
+Frequency Branch (CNN + DWT)	1.05	0.38	86.18	66.04	63.05
+Bidirectional Mamba (CNN + Mamba)	1.23	0.49	99.16	76.92	68.74
+Gated Fusion (Full FreqMamba)	1.36	0.57	99.47	77.67	69.93
Comparable-parameter CNN baseline	1.14	0.48	87.43	72.62	66.18

Note: The comparable-parameter CNN baseline is obtained by widening EfficientNet-B0 stages to match FreqMamba’s parameter count (1.14 M vs. 1.36 M). It serves to isolate the effect of model capacity.

**Table 4:** Computational efficiency and cross-domain accuracy comparison.

Model	Params (M)	FLOPs (G)	Inference Time (ms)	FPS
Xception	22.9	8.4	14.6	68
EfficientNet-B4	19.0	4.2	10.9	92
FreqMamba	1.36	0.57	5.8	172

Note: Inference time measured on NVIDIA RTX 4060 with batch size 1, averaged over 100 runs after warm-up. Xception uses 299 × 299 input, EfficientNet-B4 uses 380 × 380, and FreqMamba uses 256 × 256. Inference time measured at each model’s default input resolution. FreqMamba maintains higher accuracy while being substantially faster and lighter.

## Data Availability

The original contributions presented in this study are included in the article. Further inquiries can be directed to the corresponding author.
